# Trends and emerging frontiers of photodynamic therapy for non-melanoma skin cancer (1979–October 2024): a bibliometric analysis

**DOI:** 10.3389/fonc.2025.1580453

**Published:** 2025-07-07

**Authors:** Qi Chen, Mingming Bai, Mingmin Lu, Jingjing Chen, Shanshan Wang, Ping Sun, Linlin Li, Hong Cai

**Affiliations:** Department of Dermatology, The General Hospital of Air Force, Beijing, China

**Keywords:** non-melanoma skin cancer, photodynamic therapy, bibliometric analysis, VOSviewer, CiteSpace

## Abstract

**Introduction:**

Photodynamic therapy (PDT) is a non-invasive treatment modality for non-melanoma skin cancer (NMSC). This study aims to use bibliometric analysis to explore the research trends and development in the field of PDT for NMSC, identifying key hotspots and emerging directions.

**Methods:**

This study conducted a comprehensive literature search in the Web of Science Core Collection database and applied bibliometric analysis and visualization using tools such as VOSviewer, CiteSpace, and the R package “bibliometrix”.

**Results:**

A total of 358 publications were analyzed, with the USA led in contributions and collaborations, with Yale University being the top institution. The *Journal of the American Academy of Dermatology* made contributions to the field. Prominent authors included Richard L. Edelson, Arthur H. Rook, and Robert S. Stern. The keywords were categorized into four distinct clusters. The keywords were concentrated into four clusters: Treatment methods and targeted conditions, Cellular and immune responses, Clinical applications and therapeutic management and Techniques and research methodologies. Keywords such as “versus host disease” and “extracorporeal photopheresis” continue to emerge prominently in 2024.

**Conclusion:**

This study provides a comprehensive overview of the distribution patterns and key research hotspots in the field of PDT for NMSC. Future research trends should focus on treatment technologies and immune responses.

## Introduction

Non-melanoma skin cancer (NMSC) is a group of malignant skin tumors distinct from melanoma, with basal cell carcinoma (BCC) and squamous cell carcinoma (SCC) being the two most common types (1). Among these, BCC is the most prevalent, typically characterized by slow growth and local invasiveness, while SCC has a slightly higher potential for metastasis (2). Rare NMSC types, such as Merkel cell carcinoma, present significant clinical challenges due to their high aggressiveness (3). Globally, the incidence of NMSC has been steadily increasing, primarily attributed to increased ultraviolet exposure and the ongoing aging of the population (2). According to statistics, from 1990 to 2019, the age-standardized incidence rate of NMSC rose from 54.08 per 100,000 to 79.10 per 100,000, with an average annual growth rate of approximately 1.78% (4). Given the high incidence of NMSC, it poses a significant public health burden. In addition to the potential for disfigurement, the frequent need for treatment substantially diminishes the quality of life for patients (5). Therefore, there is an urgent need to develop effective and accessible treatment options to address this growing public health challenge (6).

Photodynamic therapy (PDT) utilizes its unique mechanism to selectively target and destroy tumor cells (7), making it an important treatment modality for NMSC. The basic principle of PDT involves the application of a photosensitizer to the affected area, which is then activated by exposure to a specific wavelength of light, leading to the production of reactive oxygen species that damage malignant cells (8). Due to its non-invasive nature and favorable cosmetic outcomes for the skin, PDT has garnered widespread attention in the treatment of skin tumors, particularly in the management of superficial NMSC, recurrent lesions, and refractory cases that do not respond well to traditional surgical treatments (9). The selective cytotoxicity of PDT minimizes damage to surrounding healthy tissues, making it especially suitable for areas where the preservation of skin aesthetics and functional integrity is critical (10). Research has shown that PDT is highly effective in treating superficial BCC and SCC, and as a well-tolerated treatment, it has been widely adopted in dermatology and oncology (11). The research conducted by L. Bernal-Masferrer et al. (12) demonstrates that 91% of cases of BCC achieved complete remission three months following photodynamic diagnosis treatment, with a sustained response rate of 76% observed five years post-treatment. Additionally, another study corroborates these findings by indicating that, in the treatment of cutaneous SCC, the integration of photodynamic diagnosis and therapy yields enhanced therapeutic outcomes and prognosis when compared to conventional surgery conducted under white light (13).

Currently, PDT is receiving increasing attention in the treatment of NMSC. However, the global research hotspots and future development trends in this field remain unclear, requiring further systematic exploration and analysis. Bibliometric analysis, through the quantitative analysis of scientific literature, can reveal the research content, current hotspots, and future trends in a specific field (14). There is a lack of bibliometric analysis on the application of PDT in NMSC. To fill this gap, this study aims to explore the research trends and developments of PDT in the NMSC field through bibliometric analysis, identifying key research hotspots and emerging directions.

## Materials and methods

### Literature search and selection

A comprehensive literature search was performed using the Web of Science Core Collection (WoSCC), a widely recognized and high-quality multidisciplinary database for scientific research indexing (15). The final search strategy was developed based on previous studies (16–19) as follows: (TS=(“NMSC*” OR NMSC OR “basal cell carcinoma*” OR “squamous cell carcinoma*” OR SCC OR “actinic keratosis*” OR “Atypical Fibroxanthoma*” OR “Sebaceous Carcinoma*” OR “Kaposi’s Sarcoma*” OR “Cutaneous T-Cell Lymphoma*” OR “*In Situ* Squamous Cell Carcinoma*” OR “Bowen′s Disease*” OR “extramammary Paget′s disease*” OR “Merkel Cell Carcinoma*”)) AND TS=((Photodynamic AND (therapy*OR treat*)) OR “Photochemotherapy” OR “Photochemotherapies”).

Data collected included publication and citation counts, titles, author details, institutions, countries/regions, keywords, and journals, all in text format, for subsequent bibliometric analysis. To maintain consistency, the search was conducted on October 9, 2024, specifically targeting English-language articles, excluding non-English and other types of publications.

### Statistical analysis and visualization

VOSviewer (v1.6.20), CiteSpace (v6.3.R1), and the R package “bibliometrix” (v4.3.3) were used for bibliometric analysis and visualization. VOSviewer mapped co-occurrences of countries, institutions, authors, and keywords, highlighting research themes and collaborations (20). Nodes represented entities, with size indicating frequency and color denoting research clusters or, for keywords, average publication year. Thicker links showed stronger co-occurrence relationships (21). CiteSpace analyzed research trend bursts from January 1994 to October 2024, using keywords as nodes and annual data slicing (22). A visual timeline highlighted the top 5 keywords per slice via combined pruning, with color gradients indicating research activity duration (23). Academic impact was assessed using the H-index (24) for author productivity and citation impact, and Journal Impact Factor (IF) from Journal Citation Reports for journal influence, reflecting average citations over two years (25).

## Results

### An overview of publications

**Figure 1:**
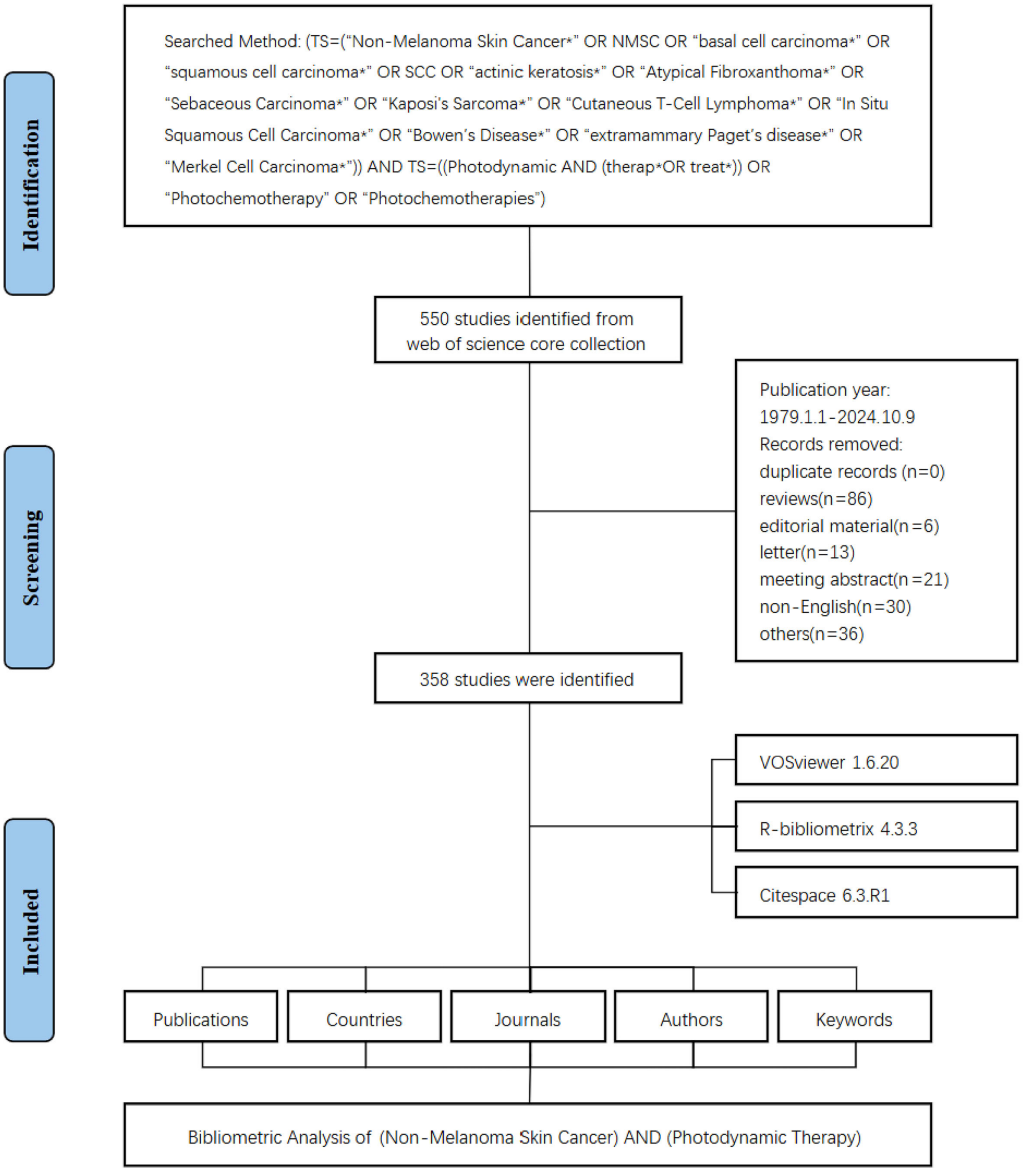
Flowchart of the literature screening process.

**Figure 2:**
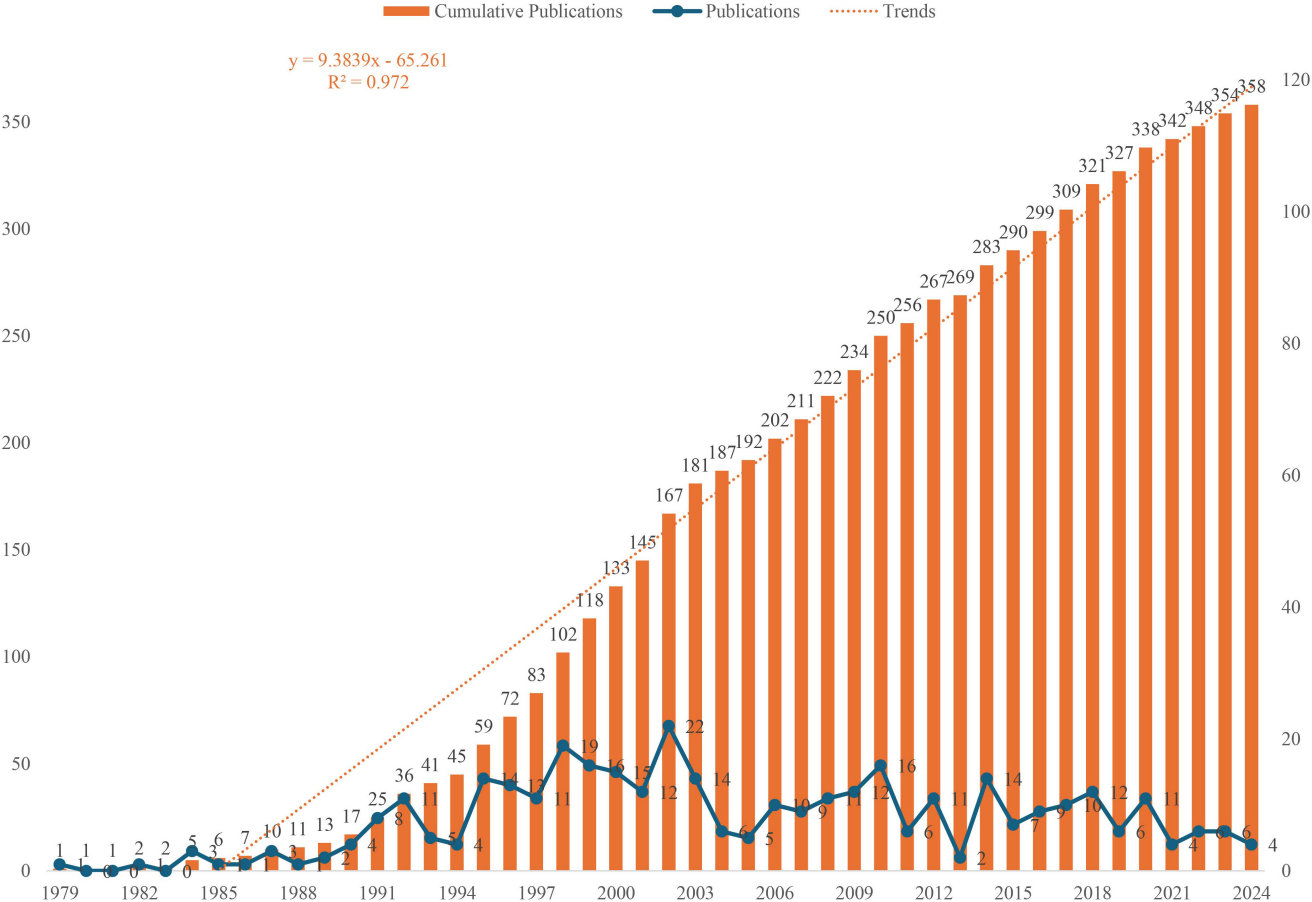
Annual number of publications on PDT for NMSC.

A total of 358 studies were identified from the WoSCC database, spanning January 1, 1979, to October 9, 2024. The detailed search workflow was shown in **Figure 1**. These studies involved 1,595 authors from 845 institutions, published across 137 journals. The annual publication trend in this research field was shown in **Figure 2**. From 1979 to 2024, the number of publications generally exhibited an upward trend, with the highest number of publications recorded in 2001, when 22 studies were published. In recent years, the number of publications has slightly declined, but research output continued. It is important to note that the data for 2024 was only available up to the search date and does not represent the full year.

#### Analysis of countries

**Figure 3:**
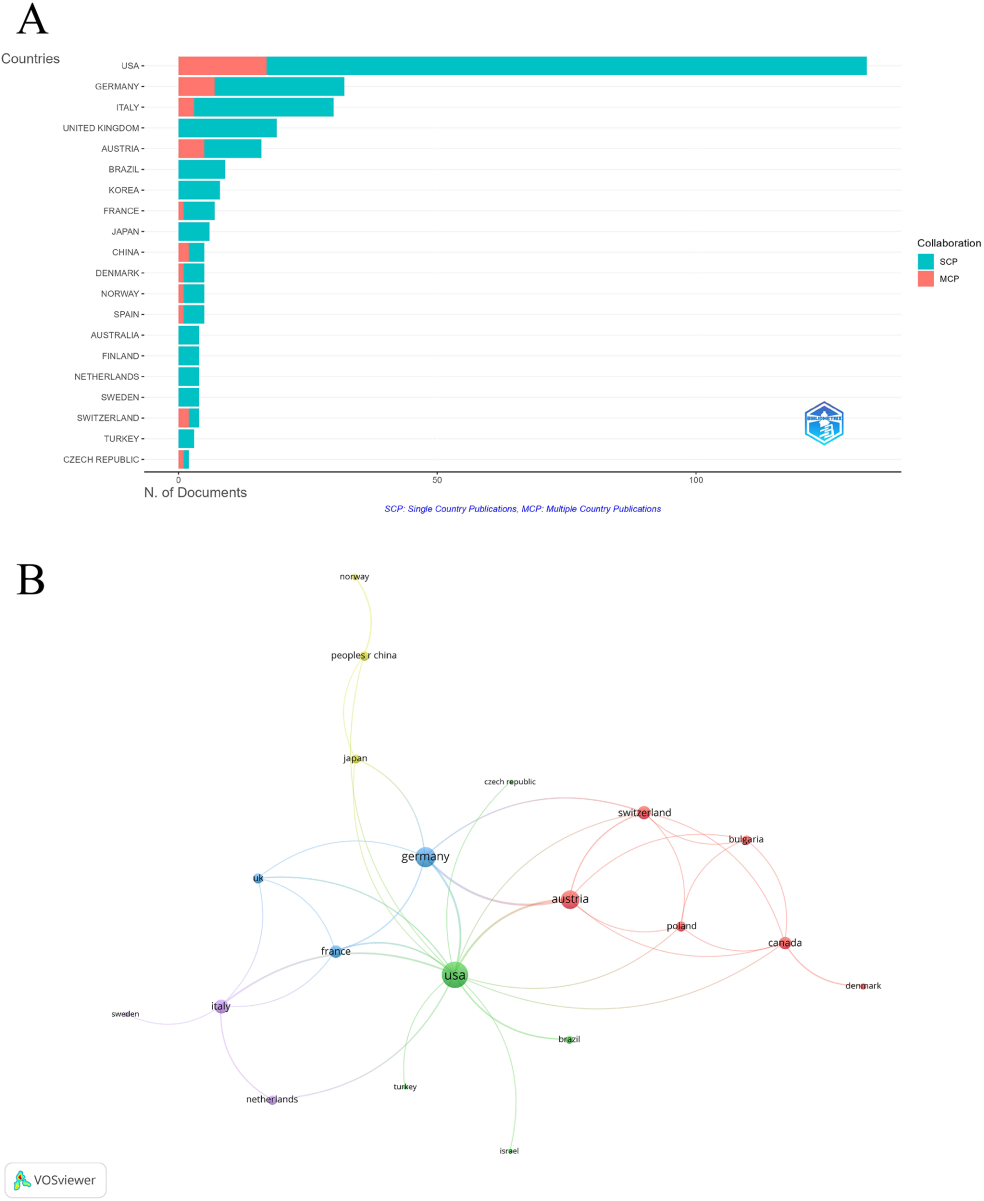
Analysis of countries. **(A)** Distribution of corresponding author’s publications by country. **(B)** Visualization map depicting the collaboration among different countries. Nodes are countries, sized by publication count. Links show co-occurrence, with thickness indicating collaboration strength. Colors represent research clusters. Link strength reflects co-occurrence frequency.

The USA (TP = 316, TC = 7,797) and Germany (TP = 102, TC = 1,012) made significant contributions to this field, ranking first and second in terms of total publications and total citations, respectively. Other countries with high influence in this field included Italy and the UK (**Table 1**; **Figure 3A**). Among the 20 countries involved in international collaborations with a minimum of two articles, the USA had the highest number of collaborations with other countries ((total link strength = 32), followed by Germany ((total link strength = 18) and Austria ((total link strength = 16) (**Figure 3B**).

**Table 1:** Publication and citation profiles of leading countries.

Country	Articles	Freq	SCP	MCP	MCP_ratio	TP	TP_rank	TC	TC_rank	Average citations
USA	133	0.372	116	17	0.128	316	1	7797	1	58.6
GERMANY	32	0.089	25	7	0.219	102	2	1012	2	31.6
ITALY	30	0.084	27	3	0.100	82	3	597	5	19.9
UNITED KINGDOM	19	0.053	19	0	0.000	33	5	882	3	46.4
AUSTRIA	16	0.045	11	5	0.313	51	4	692	4	43.2
BRAZIL	9	0.025	9	0	0.000	25	8	135	8	15
KOREA	8	0.022	8	0	0.000	14	12	81	14	10.1
FRANCE	7	0.020	6	1	0.143	25	9	101	11	14.4
JAPAN	6	0.017	6	0	0.000	20	11	96	13	16
CHINA	5	0.014	3	2	0.400	13	13	60	18	12
DENMARK	5	0.014	4	1	0.200	12	14	103	10	20.6
NORWAY	5	0.014	4	1	0.200	28	6	101	12	20.2
SPAIN	5	0.014	4	1	0.200	26	7	149	7	29.8
AUSTRALIA	4	0.011	4	0	0.000	10	17	51	20	12.8
FINLAND	4	0.011	4	0	0.000	7	22	73	16	18.2
NETHERLANDS	4	0.011	4	0	0.000	12	15	130	9	32.5
SWEDEN	4	0.011	4	0	0.000	21	10	231	6	57.8
SWITZERLAND	4	0.011	2	2	0.500	10	18	75	15	18.8
TURKEY	3	0.008	3	0	0.000	8	19	24	30	8
CZECH REPUBLIC	2	0.006	1	1	0.500	7	21	71	17	35.5

Articles: Publications of Corresponding Authors only. Freq, Frequence of Total Publications; SCP, Single Country Publications, referring to publications authored by researchers from a single country; MCP_Ratio, Proportion of Multiple Country Publications; TP, Total Publications; TP_rank, Rank of Total Publications; TC, Total Citations; TC_rank, Rank of Total Citations; Average Citations, The average number of citations per publication.

#### Analysis of institutions

**Figure 4:**
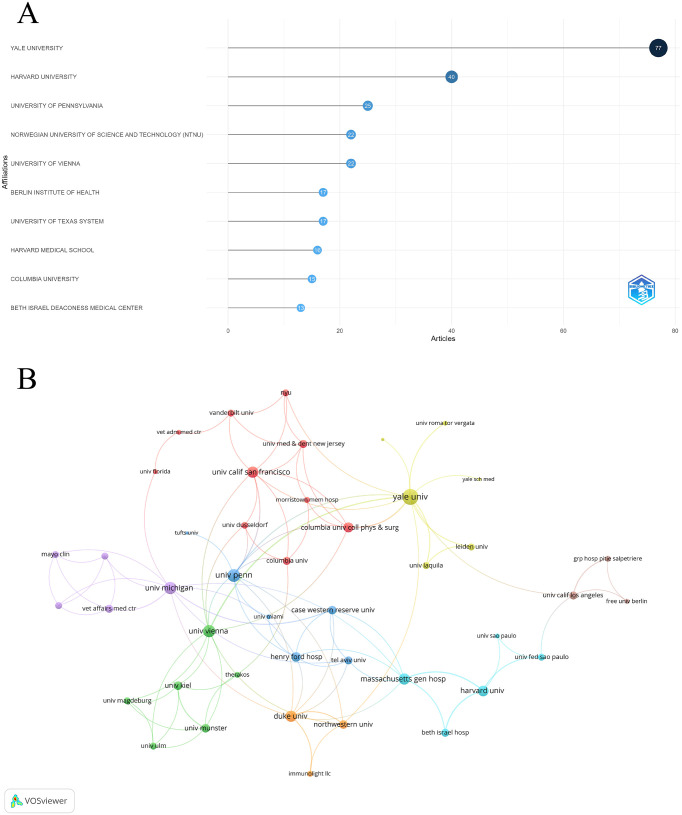
Analysis of institutions. **(A)** Top ten institutions by article count and rank. **(B)** Visualization map depicting the collaboration among different institutions. Nodes are institutions, sized by publication count. Links show co-occurrence, with thickness indicating collaboration strength. Colors represent research clusters. Link strength reflects co-occurrence frequency.

Yale University was the leading institution in PDT for NMSC research, publishing 77 articles, followed by Harvard University with 40 and the University of Pennsylvania with 25. Other significant contributors included the Norwegian University of Science and Technology and the University of Vienna, each with 22 publications (**Figure 4A**). The institutional collaboration network highlighted extensive global cooperation in this field. Yale University led with the highest number of international collaborations (total link strength = 20), followed by the University of Pennsylvania (total link strength = 13) and the University of Michigan (total link strength = 12) (**Figure 4B**).

#### Analysis of journals

**Figure 5:**
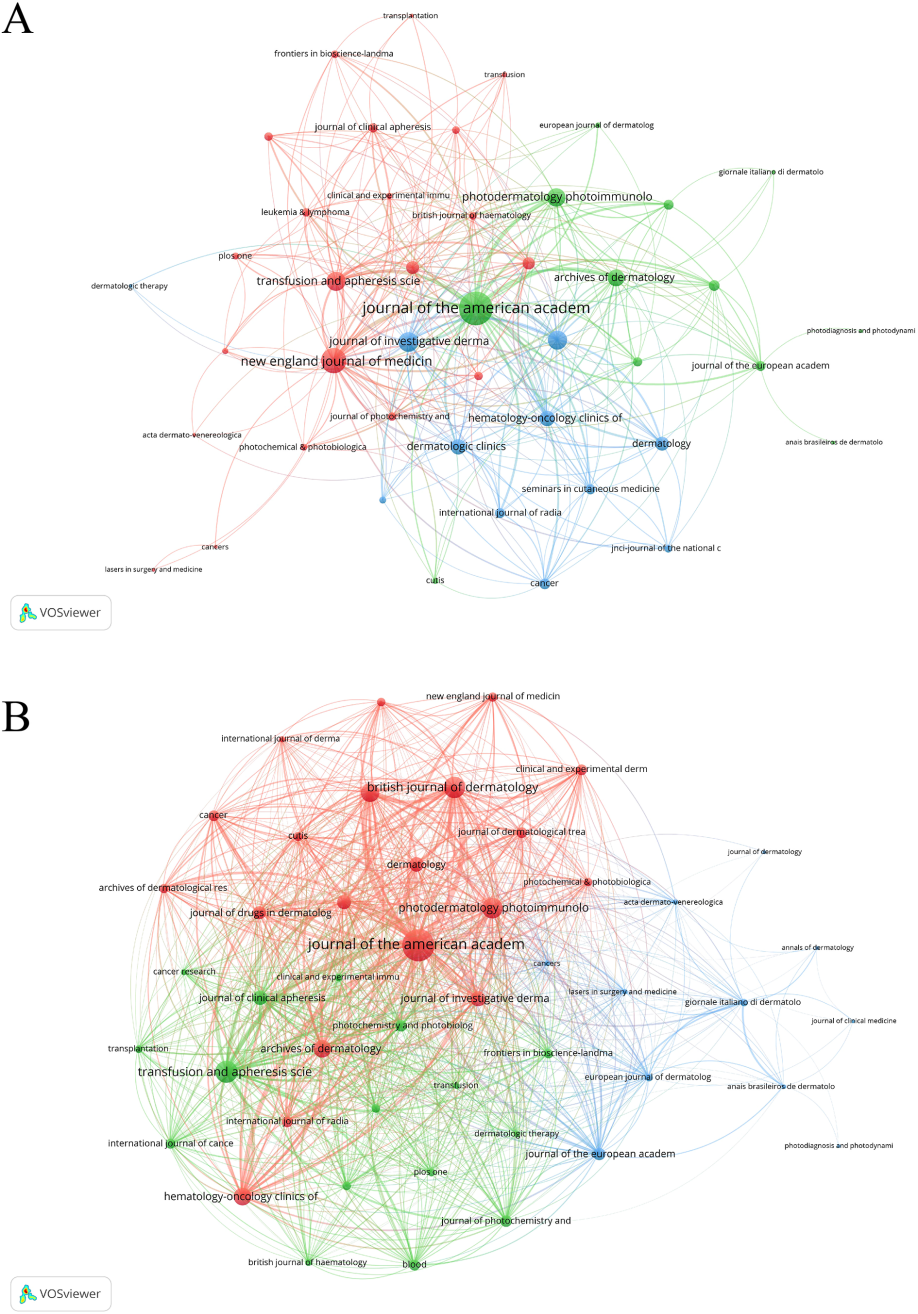
Analysis of journals. **(A)** Co-occurrence Network of Journals. Nodes are journals, sized by article count. Links show co-citation in articles, with thickness indicating strength. Colors represent thematic clusters. **(B)** Coupling Network of Journals. Nodes are journals, sized by article count. Links show co-citation in articles, with thickness indicating strength. Colors represent thematic clusters.

Among the major journals in PDT for NMSC research, the *Journal of the American Academy of Dermatology* ranked first due to its highest H-index, total publications, and total citations (H-index = 26, TP = 33, TC = 1,243). Additionally, it is worth noting that the *New England Journal of Medicine* had the highest IF, reaching 96.2 (**Table 2**). Journal co-occurrence networks, including 47 publications with at least two occurrences, identified the *Journal of the American Academy of Dermatology* (total link strength = 347), the *New England Journal of Medicine* (total link strength = 195), and the *Journal of Investigative Dermatology* (total link strength = 120) (**Figure 5A**). In coupling networks, reflecting shared references, the *Journal of the American Academy of Dermatology* led with a total link strength of 6,807, followed by *Transfusion and Apheresis Science* (3,167) and *British Journal of Dermatology* (2,970) (**Figure 5B**).

**Table 2:** Bibliometric indicators of high-impact journals.

Journal	H_index	IF	JCR_quartile	PY_start	TP	TP_rank	TC	TC_rank
JOURNAL OF THE AMERICAN ACADEMY OF DERMATOLOGY	26	12.8	Q1	1984	33	1	1243	1
BRITISH JOURNAL OF DERMATOLOGY	16	11	Q1	1987	19	2	836	2
ARCHIVES OF DERMATOLOGY	11	N/A	N/A	1992	12	5	661	4
JOURNAL OF INVESTIGATIVE DERMATOLOGY	11	5.7	Q1	1982	11	6	693	3
PHOTODERMATOLOGY PHOTOIMMUNOLOGY & PHOTOMEDICINE	11	2.5	Q2	1990	16	3	182	10
TRANSFUSION AND APHERESIS SCIENCE	9	1.4	Q4	2002	14	4	101	22
JOURNAL OF CLINICAL APHERESIS	8	1.4	Q4	1994	10	8	76	28
JOURNAL OF THE EUROPEAN ACADEMY OF DERMATOLOGY AND VENEREOLOGY	8	8.4	Q1	1997	9	9	148	13
DERMATOLOGY	7	3	Q2	1995	7	11	113	18
BLOOD	6	21	Q1	1998	6	12	467	5
DERMATOLOGIC CLINICS	6	2.2	Q2	1997	8	10	38	49
HEMATOLOGY-ONCOLOGY CLINICS OF NORTH AMERICA	6	2.5	Q3	1995	6	14	71	32
CLINICAL AND EXPERIMENTAL DERMATOLOGY	5	3.7	Q1	1984	6	13	96	23
GIORNALE ITALIANO DI DERMATOLOGIA E VENEREOLOGIA	5	N/A	N/A	2009	10	7	12	127
JOURNAL OF DERMATOLOGICAL TREATMENT	5	2.9	Q2	2000	6	15	22	79
NEW ENGLAND JOURNAL OF MEDICINE	5	96.2	Q1	1979	5	19	467	6
PHOTOCHEMISTRY AND PHOTOBIOLOGY	5	2.6	Q3	1993	5	20	224	8
ACTA DERMATO-VENEREOLOGICA	4	3.5	Q1	1992	5	16	245	7
ANAIS BRASILEIROS DE DERMATOLOGIA	4	2.6	Q2	2010	5	17	10	146
ARCHIVES OF DERMATOLOGICAL RESEARCH	4	1.8	Q3	1986	4	21	55	41

H_index, The h-index of the journal, which measures both the productivity and citation impact of the publications; IF, Impact Factor, indicating the average number of citations to recent articles published in the journal; JCR_Quartile, The quartile ranking of the journal in the Journal Citation Reports, indicating the journal’s ranking relative to others in the same field (Q1: top 25%, Q2: 25%-50%, Q3: 50%-75%, Q4: bottom 25%); TP, Total Publications; TP_rank, Rank of Total Publications; TC, Total Citations; TC_rank, Rank of Total Citations; Average Citations, The average number of citations per publication; PY_start, Publication Year Start, indicating the year the journal started publication.

#### Analysis of authors

**Figure 6:**
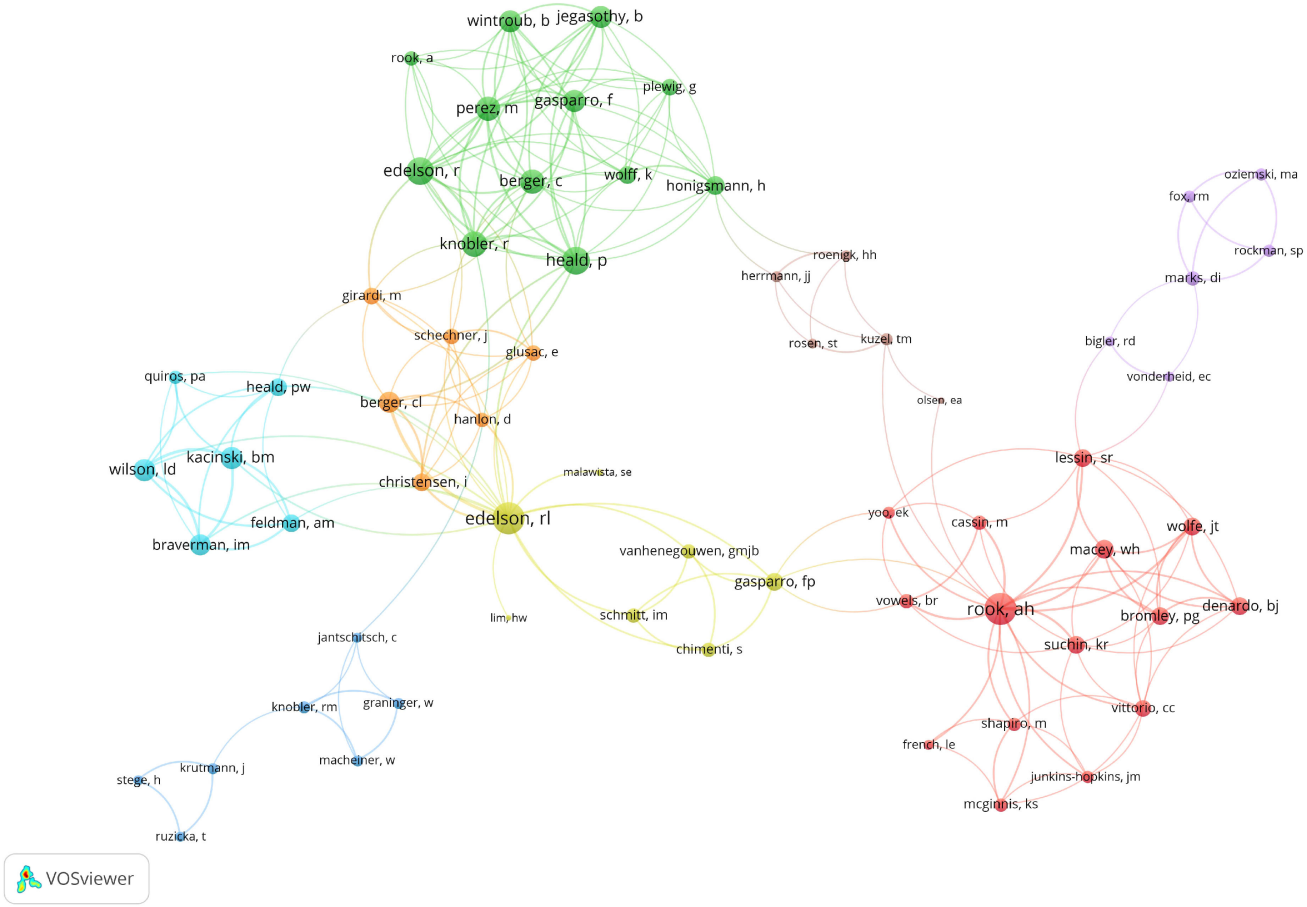
Visualization map depicting the collaboration among different authors. Nodes are authors, sized by publication count. Links show co-occurrences, with thickness indicating collaboration strength. Colors represent research clusters. Link strength reflects co-occurrence frequency.

The most influential author in this field was Richard L. Edelson, who ranked first in both H-index and total publications (H-index = 13, total publications = 15). He was followed by Arthur H. Rook (H-index = 11, total publications = 13). In terms of total citations, Robert S. Stern led with 2,111 citations (**Table 3**). Among the 64 authors involved in international collaborations with at least two articles, EDELSON, RL has the highest number of collaborations (total link strength = 46), followed by GIRARDI, MICHAEL (total link strength = 22) and ROOK, AH (total link strength = 20) (**Figure 6**).

**Table 3:** Publication and citation profiles of high-impact authors.

Authors	H_index	g-index	m-index	PY_start	TP	TP_Frac	TP_rank	TC	TC_rank
EDELSON RL	13	15	0.38	1991	15	4.47	1	755	12
ROOK AH	11	13	0.31	1989	13	3.19	2	975	11
STERN RS	9	9	0.20	1979	9	3.87	4	2111	1
EDELSON RICHARD L.	8	9	0.35	2002	9	2.17	3	337	15
GIRARDI MICHAEL	7	7	0.30	2002	7	0.94	5	262	20
BERGER CL	6	6	0.21	1996	6	1.46	7	329	17
GASPARRO FP	6	6	0.18	1992	6	1.46	8	342	14
EDELSON R	5	5	0.13	1987	5	0.84	9	1295	4
HEALD P	5	7	0.13	1987	7	1.64	6	1386	2
KACINSKI BM	5	5	0.16	1994	5	0.96	11	249	22
KNOBLER R	5	5	0.13	1987	5	1.14	12	1312	3
WILSON LD	5	5	0.16	1994	5	0.96	13	249	22
BLADON J	4	4	0.15	1999	4	2.00	14	164	28
BRAVERMAN IM	4	4	0.13	1994	4	0.63	15	217	26
DUVIC M	4	4	0.14	1996	4	1.37	16	176	27
DUVIC MADELEINE	4	4	0.24	2008	4	0.95	17	41	46
FILLER RENATA	4	4	0.27	2010	4	0.40	18	162	30
GIRARDI M	4	4	0.16	2000	4	1.23	19	106	37
HANLON DOUGLAS	4	5	0.17	2002	5	0.68	10	116	36
HONIGSMANN H	4	4	0.10	1984	4	0.75	20	1248	5

H_index, The h-index of the journal, which measures both the productivity and citation impact of the publications; g_index, The g-index of the journal, which gives more weight to highly-cited articles; m_index, The m-index of the journal, which is the h-index divided by the number of years since the first published paper; TP, Total Publications; TP_rank, Rank of Total Publications; TC, Total Citations; TC_rank, Rank of Total Citations; Average Citations, The average number of citations per publication; PY_start, Publication Year Start, indicating the year the journal started publication.

#### Analysis of publications

The most cited article in this field was titled “Treatment of cutaneous T-cell lymphoma by extracorporeal photochemotherapy. Preliminary results,” published in the *New England Journal of Medicine* (IF = 96.2) in 1987, which amassed a total of 1,031 citations. The second most cited article, “Risk of cutaneous carcinoma in patients treated with oral methoxsalen photochemotherapy for psoriasis”, was published in the *New England Journal of Medicine* in 1979 and accumulated 547 citations. The third most cited article, titled “Cutaneous squamous-cell carcinoma in patients treated with PUVA,” was also published in the *New England Journal of Medicine* in 1984 and received a total of 405 citations.

#### Analysis of keywords

**Figure 7:**
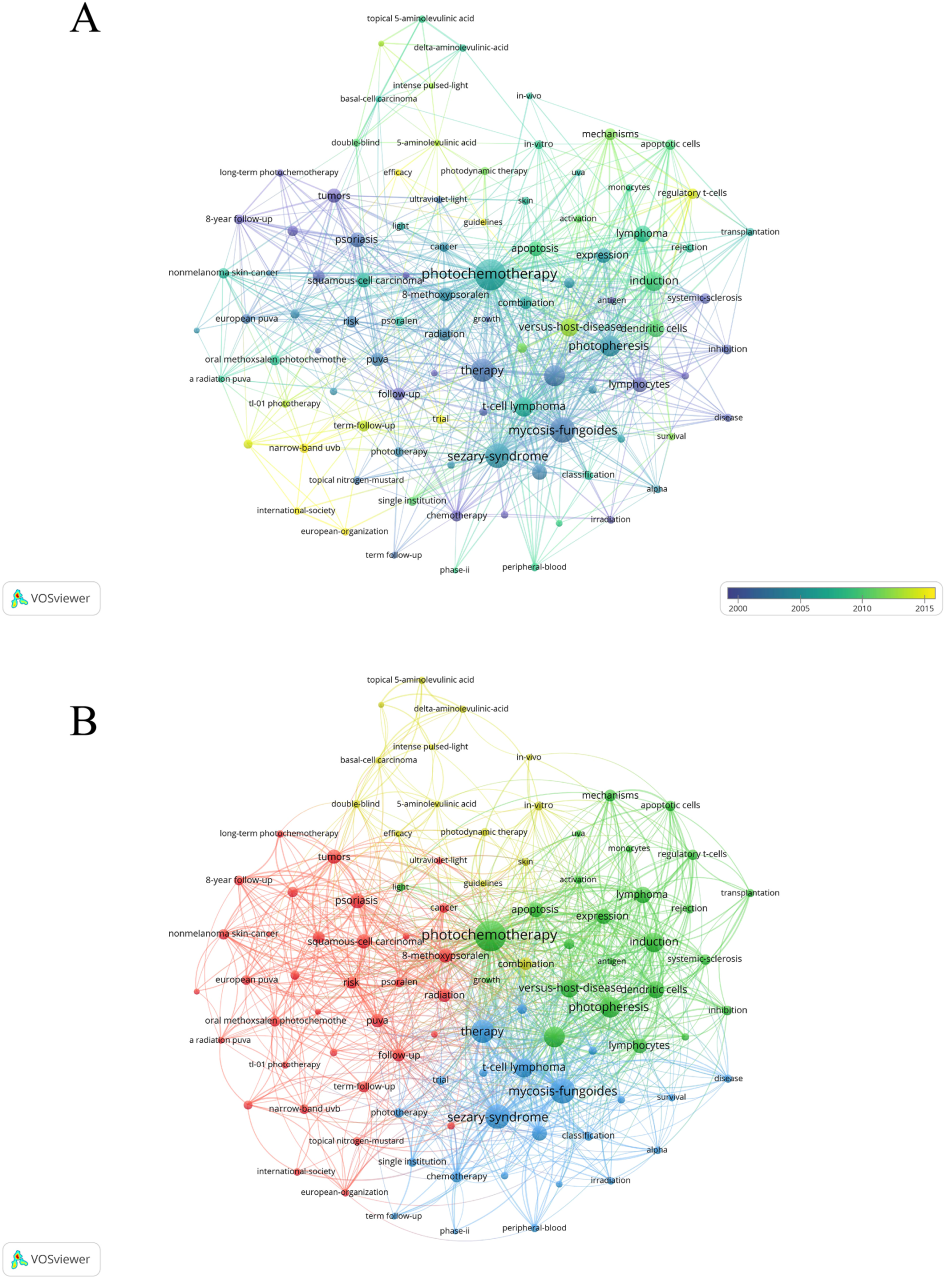
Visual analysis of keyword co-occurrence network analysis. **(A)** Visual analysis of keyword co-occurrence network (Time Trend). Nodes are keywords, sized by frequency. Links show co-occurrence in articles, with thickness indicating strength. Colors reflect average publication year (see color gradient). **(B)** Keyword Co-occurrence Network (Clusters). Nodes are keywords, sized by frequency. Links show co-occurrence in articles, with thickness indicating strength. Colors represent distinct research clusters based on thematic similarities.

VOSviewer analysis identified 96 keywords with at least 5 occurrences. Prominent keywords, such as “T-cell lymphoma” and “mycosis fungoides,” had larger nodes, indicating high frequency and strong associations (**Figure 7A**). Based on the color gradient, recent keywords like “efficacy”, “narrow-band UVB” and “regulatory T-cells” (yellow) gained prominence, while older terms like “mycosis fungoides” and “Sézary Syndrome” (purple) dominated earlier studies. Overall, the identified keywords could be categorized into four color-coded clusters (**Figure 7B**). Cluster Red: Treatment methods and targeted conditions, including terms such as “psoriasis”, “squamous-cell carcinoma” “follow-up” and “Narrow-band UVB”. Cluster Green: Cellular and immune responses, focusing on keywords like “lymphocytes”, “dendritic cells” and “regulatory T-cells”. Cluster Blue: Clinical applications and therapeutic management, with terms like “extracorporeal phototherapy”, “management” and “classification”. Cluster Yellow: Techniques and research methodologies, featuring keywords such as “5-aminolevulinic acid”, “intense pulsed light” and “efficacy”.

#### Analysis of burst keywords

**Figure 8:**
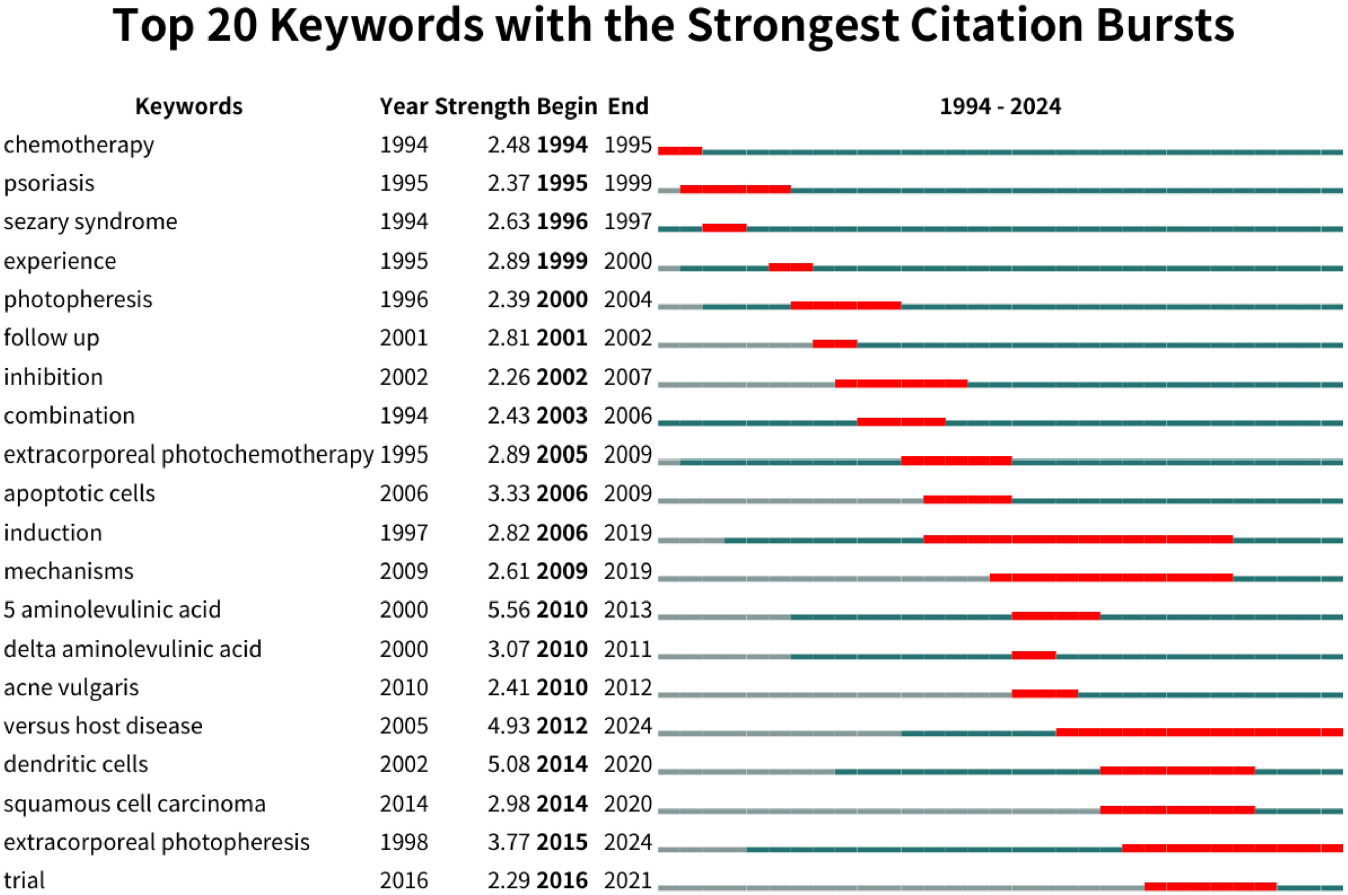
Top 20 Keywords with the Strongest Citation Bursts (CiteSpace). In the trend visualization, the blue line shows the duration of citations, and the red segment indicates the duration of citation bursts.

The analysis of the top 20 keywords with the strongest citation bursts between 1994 and 2024 revealed notable patterns in research focus and emerging trends. The keyword with the highest burst strength was “5-aminolevulinic acid” (Strength = 5.56) which peaked between 2010 and 2013. Other notable keywords with significant bursts included “dendritic cells” (Strength = 5.08), “graft-versus-host disease” (Strength = 4.93), and “extracorporeal photopheresis” (Strength = 3.77). Early bursts included keywords like “chemotherapy” (1994–1995) and “psoriasis” (1995–1999). More recent bursts continuing through 2024 were associated with keywords such as “versus host disease” and “extracorporeal photopheresis” (**Figure 8**).

## Discussion

This bibliometric study provides an unprecedented comprehensive overview of the distribution patterns and key research hotspots in the field of PDT for NMSC. A total of 1,595 authors from 845 institutions contributed to these 358 publications, which were published across 137 different journals. The USA has been a leading force in PDT research for NMSC, both in terms of publication volume and citation impact. Prominent institutions like Yale University, Harvard University, and the University of Pennsylvania have been central to this research, producing a substantial body of influential work. This leadership is a reflection of the country’s robust healthcare system and research infrastructure (26, 27). However, the USA tends to have fewer international collaborations compared to the number of independent publications. In contrast, European nations such as Germany, Italy, and the UK have made notable contributions, with Germany forming strong global research partnerships. Moving forward, enhancing collaboration between the USA and European research hubs will be crucial for further advancing the field.

The leading journal in this field is the *Journal of the American Academy of Dermatology*, with the highest number of publications, followed by the *British Journal of Dermatology* and *Photodermatology, Photoimmunology & Photomedicine*. These journals are influential in dermatologic oncology, focusing on the mechanisms (28), clinical efficacy (29), and applications (30) of PDT in treating BCC, SCC, and other types of NMSC. Richard L. Edelson was identified as a leading author in the field, distinguished by having the highest H-index and TP. He focused on investigating the immunological mechanisms of extracorporeal photochemotherapy (31), its role in modulating immune responses (32), and its therapeutic applications in cancer and immune-related conditions (33).

Highly cited studies in this field are primarily published in high-impact journals, such as the *New England Journal of Medicine*. The most-cited article, by Edelson et al., demonstrated that extracorporeal photochemotherapy effectively treated patients with disseminated cutaneous T-cell lymphoma, showing substantial efficacy in cases resistant to traditional therapies and fewer side effects (34). However, despite being published in a high-impact journal and receiving extensive citations, the study still has some limitations. The sample size was relatively small, with only 37 patients included, which may limit the generalizability of the findings. Additionally, the long-term effects and durability of the treatment were not fully addressed, and further studies with larger patient cohorts and longer follow-up periods are needed to confirm the sustained efficacy and safety of extracorporeal photochemotherapy.

### Research hotspots and frontiers

Keywords were divided into four main clusters: Cluster Red: Treatment methods and targeted conditions, Cluster Green: Cellular and immune responses, Cluster Blue: Clinical applications and therapeutic management and Cluster Yellow: Techniques and research methodologies. These keywords in the co-occurrence analysis can show the main research directions and hot topics of researchers in this field.

### Cluster red: treatment methods and targeted conditions

Keywords such as “psoriasis”, “squamous-cell carcinoma” “follow-up” and “Narrow-band UVB” underscored a focus on research and treatment of various NMSC and related dermatological conditions. A recent review suggested that PDT offers a promising non-surgical alternative for keratinocyte carcinoma (35). However, PDT may also entail adverse effects in NMSC treatment. A five-year single-center retrospective analysis (36) conducted a single-center retrospective analysis over five years involving 200 psoriasis patients treated with either phototherapy or anti-TNFα therapy. It concluded that patients undergoing phototherapy had a higher risk of developing NMSCs, emphasizing the need for closer monitoring, especially for those with scalp psoriasis. Additionally, a case report indicated that cumulative ultraviolet exposure from narrow-band UVB therapy remains a potential risk factor for NMSC (37). As treatment advances, new effective options are emerging. For instance, the mTORC1 inhibitor rapamycin (Sirolimus, SRL) has been shown to enhance PDT efficacy in treating cutaneous SCC as a pretreatment strategy (38). Furthermore, light-triggered nanomaterials demonstrate significant potential in PDT for skin cancer (39).

### Cluster green: cellular and immune responses

Keywords like “lymphocytes”, “dendritic cells” and “regulatory T-cells” highlighted the cellular mechanisms and immune modulation influenced by PDT, reflecting a strong interest in understanding the biological effects of this therapy. For instance, research (40) used flow cytometry and immunohistochemistry to analyze T cell subpopulations in BCC, SCC, and normal skin. It concluded that NMSCs exhibited a Th1-preponderant immune response with Th17 enrichment, supporting immune-based treatments and identifying Th17-mediated inflammation as a potential therapeutic target. Similarly, another study (41) employed murine models of UVB-induced actinic keratosis and SCC to analyze immune responses after PDT with or without vitamin D. It found that the combination of vitamin D and PDT enhanced immune cell recruitment and modulated immune responses, improving the efficacy of PDT for NMSCs.

### Cluster blue: clinical applications and therapeutic management

Keywords including “extracorporeal phototherapy”, “management” and “classification” reflected efforts to optimize clinical practice, evaluate therapeutic outcomes, and categorize patient responses within the clinical setting. Sarah I. Jawed and colleagues (42) conducted a systematic review on the prognosis, management, and future directions of primary cutaneous T-cell lymphoma. The review found that PDT is effective in the early treatment of related diseases and that combining PDT with low-dose bexarotene can achieve better prognostic outcomes. Research by Nan Yang et al. (13) demonstrated that PDD-guide resection combined with PDT offered a highly safe and effective treatment strategy for SCC. This approach accurately delineated tumor margins, guiding precise surgical resection. For large, multifocal, or invasive tumors located in cosmetically or functionally sensitive areas such as the scalp or face, conventional surgery may result in significant functional or aesthetic deficits. In such cases, PDT provided a reliable, minimally invasive alternative. Given its proven efficacy and minimal invasiveness, ALA-PDT hold significant promise as an adjuvant treatment for NMSCs. This was particularly relevant for large, invasive, or multifocal tumors, or those in sensitive areas, as well as for patients with advanced age, comorbidities, limited surgical tolerance, or concerns about postoperative quality of life.

### Cluster yellow: techniques and research methodologies

Keywords such as “5-aminolevulinic acid”, “intense pulsed light” and “efficacy” emphasized the technical core and practical application outcomes of the PDT. For example, Zhao et al. (43) conducted a retrospective study comparing three injection methods of 5-aminolevulinic acid in NMSC patients. They concluded that needle-free injection followed by red light PDT achieved higher treatment response with fewer adverse effects compared to conventional and plum-blossom needle injections. As research progresses, researchers continue to explore new materials for PDT applications. For example, studies have found that pyrene-based photosensitizers exhibit strong anti-tumor activity, low toxicity, and high skin retention, making them a promising candidate for PDT photosensitizers (44). It is noteworthy that, in recent years, a growing body of research has concentrated on the integration of PDT and immunotherapy for the treatment of NMSC, including cutaneous SCC. Specifically, the combination of PDT and immunotherapy has demonstrated promising therapeutic potential, particularly in the management of malignant ulcers associated with cutaneous SCC (45). This observation suggests that the future integration of PDT with immunotherapy may assume a significant role in the treatment of these malignancies.

The ongoing emphasis on “versus host disease” and “extracorporeal photopheresis” suggests that future research trends in NMSC PDT treatment may focus on immunological aspects and targeted applications. The keyword “extracorporeal photopheresis” (ECP) experienced a steady citation burst from 2015 to 2024, reflecting a rising trend in utilizing PDT for immunologically complex conditions. ECP applications extended beyond skin cancers to immune-related diseases like Graft-versus-host disease (46). This versatility highlighted PDT’s potential to modulate immune responses in systemic conditions. Research during this period refined ECP techniques to enhance specificity and efficacy in immune cell targeting, broadening its applications in oncology and immunotherapy. Investigators also explored the cellular mechanisms by which ECP modulated immune functions, aiming to expand its therapeutic utility in managing immune-mediated conditions. The keyword “versus host disease” (GVHD) remained prominent, with a citation burst extending from 2012 to 2024, demonstrating sustained interest in using PDT to manage immune complications (47). GVHD posed significant challenges in transplantation, and researchers investigated PDT as a less toxic alternative to traditional immunosuppressive therapies. Studies focused on understanding how PDT and ECP interacted with immune cells to safely manage GVHD while reducing immunosuppression and improving patient outcomes.

Overall, these trends highlighted a growing emphasis on immunological and mechanistic studies in PDT. Efforts aimed to enhance treatment efficacy, minimize side effects, and develop personalized therapies for NMSC and related immune conditions.

### Strengths and limitations

This bibliometric study provides the first comprehensive analysis of distribution trends and key research focuses in PDT for NMSC. A major strength of this study is its multi-decade analysis, which highlights influential literature and evolving research themes in this field. However, like other bibliometric studies, there are limitations. The reliance on citation counts may not fully reflect an article’s clinical impact or relevance. Furthermore, the study’s dependence on WoSCC as the sole database may introduce selection bias, as it may exclude relevant studies indexed in other databases, such as PubMed or Scopus, which could provide a broader perspective on PDT research. Additionally, restricting the analysis to English-language publications may limit the scope, potentially overlooking significant studies in other languages. To address these limitations, future research could integrate multiple databases to ensure a more comprehensive literature coverage and consider multilingual studies to capture a wider range of contributions. Specific directions for future research include exploring immune mechanisms underlying PDT efficacy, such as the role of immune checkpoint inhibitors, and advancing technological improvements, such as optimizing light delivery systems or developing novel photosensitizers, to enhance treatment outcomes.

## Conclusion

This study provides a comprehensive overview of the distribution patterns and key research hotspots in the field of PDT for NMSC. It highlights the significant advancements made in treatment strategies and identifies emerging areas of interest. Future research trends should focus on enhancing treatment technologies, improving efficacy, and exploring the immunological mechanisms underlying PDT to optimize therapeutic outcomes.

## Data Availability

The original contributions presented in the study are included in the article/supplementary material. Further inquiries can be directed to the corresponding author.
